# The impact of diabetes on multiple avoidable admissions: a cross-sectional study

**DOI:** 10.1186/s12913-019-4840-4

**Published:** 2019-12-27

**Authors:** Joana Seringa, Ana Patrícia Marques, Bruno Moita, Cátia Gaspar, João Filipe Raposo, Rui Santana

**Affiliations:** 1 NOVA National School of Public Health, Universidade NOVA de Lisboa, Lisbon, Portugal; 2 NOVA National School of Public Health, Public Health Research Centre, Universidade NOVA de Lisboa, Lisbon, Portugal; 3 Comprehensive Health Research Center (CHRC), Lisbon, Portugal; 4 Algarve University Hospital Center, Faro, Portugal; 5 NOVA Medical School, Universidade NOVA de Lisboa, Lisbon, Portugal; 6 Associação Protectora dos Diabéticos de Portugal, Lisbon, Portugal

**Keywords:** Multiple admissions for ACSC, Diabetes, Multimorbidity

## Abstract

**Background:**

Multiple admissions for ambulatory care sensitive conditions (ACSC) are responsible for an important proportion of health care expenditures. Diabetes is one of the conditions consensually classified as an ACSC being considered a major public health concern. The aim of this study was to analyse the impact of diabetes on the occurrence of multiple admissions for ACSC.

**Methods:**

We analysed inpatient data of all public Portuguese NHS hospitals from 2013 to 2015 on multiple admissions for ACSC among adults aged 18 or older. Multiple ACSC users were identified if they had two or more admissions for any ACSC during the period of analysis. Two logistic regression models were computed. A baseline model where a logistic regression was performed to assess the association between multiple admissions and the presence of diabetes, adjusting for age and sex. A full model to test if diabetes had no constant association with multiple admissions by any ACSC across age groups.

**Results:**

Among 301,334 ACSC admissions, 144,209 (47.9%) were classified as multiple admissions and from those, 59,436 had diabetes diagnosis, which corresponded to 23,692 patients. Patients with diabetes were 1.49 times (*p* < 0,001) more likely to be admitted multiple times for any ACSC than patients without diabetes. Younger adults with diabetes (18–39 years old) were more likely to become multiple users.

**Conclusion:**

Diabetes increases the risk of multiple admissions for ACSC, especially in younger adults. Diabetes presence is associated with a higher resource utilization, which highlights the need for the implementation of adequate management of chronic diseases policies.

## Key points

- Increased costs and length of stay on multiple admissions for ACSC where diabetes diagnosis is present;
- Increased risk of multiple admissions for ACSC by the presence of diabetes, and especially at younger adults with diabetes;
- The need to improve the management of chronic diseases through a set of strategies, such as health promotion and health education.

## Background

Ambulatory Care Sensitive Conditions (ACSC) are defined as a group of medical conditions for which adequate ambulatory care can, potentially, prevent the need for hospital admission or the worsening of complications [1–5]. Admissions for ACSC are associated with worse quality of ambulatory care and represent a significant burden on health care systems and a negative experience to patients [6–8].

Within this concept, the subgroup of multiple admissions for ACSC has been gaining a growing importance. Multiple admissions for ACSC are characterized by the frequent utilization of inpatient care for ACSC by the same patient within a period of time [9]. While readmission assessment is mainly focused on quality of inpatient and transitional care [10], the appraisal of multiple admissions allows the assessment of ambulatory care quality before and after discharge [9, 11]. The occurrence of ACSC admissions is worrying and may highlight the need for specific interventions in order to correct the underlying processes of care, but the recurrence over time of this admissions may indicate systemic problems addressing patients’ health needs.

Among other reasons, the presence of multiple chronic conditions challenges ambulatory care and increases the likelihood of admission for ACSC [12, 13] and is associated with higher costs and healthcare utilization.

Diabetes is one of the most prevalent chronic disease worldwide with a strong growth trend [14–16]. A large number of diabetes-related comorbidities, such as cardiovascular diseases, nephropathy and depression are documented in the literature [17, 18] as well as multimorbidity in patients with diabetes. In Portugal, diabetes is the sixth most frequent ACSC main cause of admission, representing 3.9% of the total ACSC admissions [19]. Although it is not the most frequent ACSC, as principal diagnosis, diabetes may contribute to aggravate other chronic diseases, such as Chronic Obstructive Pulmonary Disease (COPD) [20] and congestive heart failure [21].

Diabetes requires adequate management, as to avoid serious micro and macrovascular complications with a huge impact on the quality of life to the individuals and a significant burden on healthcare and global economic systems [22]. Diabetes is associated with an increase in healthcare costs related either to a rising number of admissions and consumption of other medical resources or to absenteeism and loss of productivity [15]. However, the impact of diabetes on the occurrence of multiple admissions for ACSC has been neglected in previous studies. The aim of this study was to investigate the impact of diabetes on the occurrence of multiple admissions for ACSC.

## Methods

### Data sources and selection criteria

Data on inpatient admissions in all public Portuguese National Health Service (NHS) hospitals from 2013 to 2015 (*n* = 3,041,447) was used. This database contains a summary of each inpatient admission, including demographic and clinical characteristics, such as age, sex, diagnosis and procedures coded according with International Classification of Diseases, Version 9 – Clinical Modifications (ICD-9-CM). A unique anonymized patient identifier allowed the linkage of all admissions for each patient in any of the public hospitals.

We excluded admissions from individuals aged younger 18 years old, admissions to specialized hospitals and admissions with incomplete records (error diagnosis-related group, missing gender and missing patient identifier). Pregnancy childbirth and puerperium admissions, radiotherapy procedures, haemodialysis diagnosis on patients with chronic renal failure and patients with more than thirty admissions were also excluded, accounting for a total of 1,071,603 excluded admissions. The final sample contained 1,969,844 admissions associated to 1,220,363 distinct patients.

### Variables

We defined multiple admissions for ACSC as the outcome measure of our study. An indicator variable assuming value 1 was created if, over the three years considered, the patient had more than one admission for any ACSC.

ACSC were identified by the Prevention Quality Indicators (PQIs) methodology defined by the Agency for Healthcare Research and Quality (AHRQ) [23]. PQIs methodology is based on a review and selection process that has made it a standard in this area of research [24]. This methodology also allows a detailed analysis of admissions for ACSC related to diabetes since four of the PQIs are directly related to this disease. We used the PQI 90 Overall composite that includes 11 validated PQIs for the adult population (Additional file 1: for PQI 90 details) [23].

A diabetes case was defined if any diagnosis within category 250 from ICD-9-CM was present. Risk associated to comorbidities was assessed using an enhanced version of Charlson Comorbidity Index (CCI) [25]. Costs per admission were estimated using the Diagnosis Related Groups (DRG) Portuguese NHS prices defined for the analysed years [26, 27].

### Statistical analysis

We characterized multiple admissions for ACSC by sex, age group, type of admission, CCI, ACSC cause and estimated the average length of stay and the cost per admission comparing two distinct groups: admissions with and without diabetes. Chi-square test was used to compare proportions between groups. Mann-Whitney test was used to compare the average length of stay and estimated unit cost per admission in both groups.

Logistic regression was performed to assess the association between the chance of multiple admissions for any ACSC and the presence of diabetes adjusting for age and sex. In this baseline model (model 1), odds ratio estimates should be interpreted as the increased chance of multiple admissions for ACSC if a patient has diabetes, controlling for patients’ age and sex.

To test for the hypothesis that the association between diabetes and the chance of multiple admissions by any ACSC is not constant across the age groups, an interaction term between diabetes and age was computed and modelled in the regression analysis. In this model (model 2), diabetes odds ratio should be interpreted as the increased chance of multiple admissions difference in patients at age reference category (18–39 years old). Simultaneously, age group odds ratio should be interpreted as the increased chance of multiple admissions to reference category age group if patients doesn’t have diabetes (diabetes = 0). To obtain the increased chance of multiple admissions for any ACSC in a specific age group between patients with and without diabetes, interaction coefficient should be multiplied by diabetes coefficient.

Given that AHRQs methodology for classification of ACSC admissions included 4 out of 11 PQIs directly related to diabetes, and assuming the hypothesis that our results could have been influenced and overestimated by the model itself, a sensitive analysis was performed to test the risk of multiple admissions by the presence of diabetes on admissions whose main diagnosis of admission was not diabetes. We chose to analyse bacterial pneumonia, heart failure and urinary tract infections (UTI) since these three conditions were the most frequent cause of admission in our sample.

Statistical analyses were performed using SPSS software version 24.0. A level of significance < 0.05, for a 95% confidence interval, was defined.

## Results

**Fig. 1:**
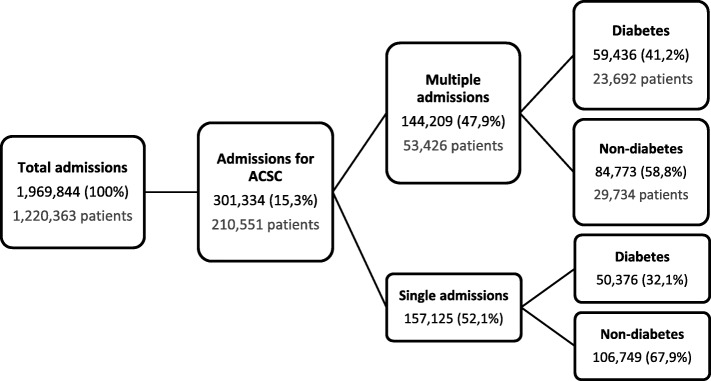
Diagram of distribution of admissions, patients and total costs

In this 3 year period, ACSC represented 15.3% of all admissions occurred at all public Portuguese NHS hospitals, corresponding to a financial burden of 710.023.509€. Approximately 48% of these admissions were considered multiple admissions for ACSC and, of those, 41% had a diabetes diagnosis, which corresponded to 59,436 admissions and 23,692 patients. Figure 1 illustrates the distribution of the number of admissions and the number of patients, separating single admissions and multiple admissions and subdividing by the presence or absence of diabetes.

Multiple admissions were more frequent in women (50.7%), in patients with 65 years old or older (86.3%) and in patients with CCI score of 1, 2 or 3 (65.2%). These admissions were mainly unplanned (95.4%). Bacterial pneumonia was responsible for 30.3% of multiple admissions followed by heart failure and UTI that represented 26.6 and 16.8% of multiple admissions, respectively. These results are summarized in Table 1, which also shows results comparing multiple admissions with and without diabetes diagnosis. A higher proportion of women was found in admissions where diabetes diagnosis was present either as principal or secondary cause of admission. Multiple admissions with diabetes diagnosis had a higher proportion of CCI with a score 2 (24.1%) while in those without diabetes the score 1 was the most frequent (32.2%). Moreover, in patients with diabetes, a score higher or equal to 7 was greater (5.4%), when compared to patients without diabetes. Patients aged 80 years old or older represented 44% of cases and 93% of admissions were classified as unplanned admissions. Among diabetic multiple users, 65% of admissions were due to bacterial pneumonia, heart failure and UTI whereas diabetes as the principal cause of admission represented only 23% of admissions.

**Table 1:** Descriptive statistics: multiple admissions for ACSC with and without diabetes

	Multiple Admissions for ACSC					
	Diabetes *N* (%)		Without Diabetes *N* (%)		Total *N* (%)	
Total Number	59,436	(41.2%)	84,773	(58.8%)	144,209	(100%)
Sex^a^						
Female	31,337	(52.7%)	41,825	(49.3%)	73,162	(50.7%)
Male	28,099	(47.3%)	42,948	(50.7%)	71,047	(49.3%)
Age Group^a^						
18–39	1187	(2.0%)	1552	(1.8%)	2739	(1.9%)
40–64	8256	(13.9%)	10,273	(12.1%)	18,529	(12.8%)
65–79	23,854	(40.1%)	24,118	(28.5%)	47,972	(33.3%)
≥ 80	26,139	(44.0%)	48,830	(57.6%)	74,969	(53.0%)
Admission Type^a^						
Planned	3794	(6.4%)	2763	(3.3%)	6557	(4.5%)
Unplanned	55,514	(93.4%)	82,003	(96.7%)	137,517	(95.4%)
Others^b^	128	(0.2%)	7	(0%)	135	(0.1%)
CCI^a^						
0	0	(0.0%)	11,431	(13.5%)	11,431	(7.9%)
1	6413	(10.8%)	27,273	(32.2%)	33,686	(23.4%)
2	14,342	(24.1%)	20,859	(24.6%)	35,201	(24.4%)
3	12,476	(21.0%)	12,673	(14.9%)	25,149	(17.4%)
4	9869	(16.6%)	7388	(8.7%)	17,257	(12.0%)
5	8401	(14.1%)	2541	(3%)	10,942	(7.6%)
6	4698	(7.9%)	1349	(1.6%)	6047	(4.2%)
≥ 7	3237	(5.4%)	1259	(1.5%)	4496	(3.1%)
ACSC Cause^a^						
Bacterial Pneumonia	13,645	(23.0%)	29,983	(35.4%)	43,628	(30.3%)
Heart Failure	16,473	(27.7%)	21,820	(25.7%)	38,293	(26.6%)
UTI	9220	(15.5%)	15,053	(17.8%)	24,273	(16.8%)
COPD or Asthma in Older Adults	5140	(8.6%)	14,195	(16.7%)	19,335	(13.4%)
Diabetes long-term complications	8402	(14.1%)	0	(0.0%)	8402	(5.8%)
Dehydration	1262	(2.1%)	2325	(2.7%)	3587	(2.5%)
Diabetes short-term complications	2000	(3.4%)	0	(0.0%)	2000	(1.4%)
Lower-Extremity Amputation among Patients with Diabetes	2330	(3.9%)	0	(0.0%)	2330	(1.6%)
Uncontrolled Diabetes	1139	(1.9%)	0	(0.0%)	1139	(0.8%)
Hypertension	940	(1.6%)	1157	(1.4%)	2097	(1.5%)
Asthma in Younger Adults	3	(0.0%)	240	(0.3%)	243	(0.2%)

^a^Applied Chi-Square test. Difference between groups statistically significant (*p* ≤ 0.001)

^b^Includes: 1 episode of private medicine and 134 episodes of Integrated Management System of Subscribers for Surgery (SIGIC)

Table 2 summarizes the distribution of multiple admissions by cause of ACSC and by age categories. Causes of multiple admissions differ by age categories and by the presence of diabetes. Diabetic patients aged 18 to 39 years old causes of multiple admissions were driven by diabetes short and long-term complications that represented together 80.4% of admission in this age category. Non-diabetic patients aged 18 to 39 years main causes of admission were UTI (43.9%) and bacterial pneumonia (27.9%). Different distribution of causes of multiple admissions was also observed in patients aged 40 to 64 years old when comparing patients with and without diabetes. 66.6% patients without diabetes were admitted due to COPD (33.9%) and bacterial pneumonia (26.7%) whereas 63.7% patients with diabetes were admitted due to long term diabetes complications (32.1%) heart failure (19.6%) and bacterial pneumonia (12.0%). In the age categories of 65 to 79 years old and 80 years old and above more than 50% of multiple admissions in both groups were concentrated in two main causes namely heart failure and bacterial pneumonia.

**Table 2:** Distribution of multiple admissions by cause of ACSC and by age categories between episodes with and without diabetes

ACSC cause	18–39 years				40–64 years				65–79 years				80 or more years			
	Diabetes		Non-Diabetes		Diabetes		Non-Diabetes		Diabetes		Non-Diabetes		Diabetes		Non-Diabetes	
	(#)	%	(#)	%	(#)	%	(#)	%	(#)	%	(#)	%	(#)	%	(#)	%
Diabetes short-term complications	566	47.4%	0	0.0%	529	6.2%	0	0.0%	439	1.8%	0	0.0%	466	1.8%	0	0.0%
Diabetes long-term complications	394	33.0%	0	0.0%	2732	32.1%	0	0.0%	3459	14.2%	0	0.0%	1817	6.9%	0	0.0%
COPD or asthma in older adults	0	0.0%	0	0.0%	767	9.0%	3484	33.9%	2428	10.0%	5488	22.8%	1945	7.3%	5223	10.7%
Hypertension	3	0.3%	39	2.5%	121	1.4%	172	1.7%	384	1.6%	305	1.3%	432	1.6%	641	1.3%
Heart failure	16	1.3%	133	8.6%	1673	19.6%	1841	17.9%	7342	30.2%	6465	26.8%	7442	28.1%	13,381	27.4%
Dehydration	8	0.7%	25	1.6%	85	1.0%	109	1.1%	426	1.8%	492	2.0%	743	2.8%	1699	3.5%
Bacterial pneumonia	44	3.7%	433	27.9%	1024	12.0%	2739	26.7%	4880	20.0%	7650	31.7%	7697	29.0%	19,161	39.2%
UTI	79	6.6%	682	43.9%	827	9.7%	1928	18.8%	3581	14.7%	3718	15.4%	4733	17.9%	8725	17.9%
Uncontrolled diabetes	70	5.9%	0	0.0%	243	2.9%	0	0.0%	382	1.6%	0	0.0%	444	1.7%	0	0.0%
Asthma in younger adults	3	0.3%	240	15.5%	0	0.0%	0	0.0%	0	0.0%	0	0.0%	0	0.0%	0	0.0%
Lower-extremity amputation among patients with diabetes	11	0.9%	0	0.0%	516	6.1%	0	0.0%	1019	4.2%	0	0.0%	784	3.0%	0	0.0%

As shown in Table 3, admissions with diabetes had a higher average of length of stay (11.1 ± 12.4 days) and a higher average unit cost of the admission (2543 ± 2797€) when compared to admissions without diabetes (10.0 ± 9.3 days) and (2261 ± 2135€), respectively. These differences were found to be statistically significant accordingly with Mann-Whitney Test (*p* < 0.001).

**Table 3:** Comparison between multiple admissions for ACSC, with and without diabetes, regarding the length of stay and estimated unit cost per admission

Multiple admissions for ACSC		Diabetes	Non-diabetes
Average length of stay (days)^a^	Mean	11.10	10.00
	Median	8.0	8.0
	Standard deviation	12.42	9.30
	Range	0–700	0–266
Estimated unit cost per admission (€)^a^	Mean	2543	2261
	Median	1938	1938
	Standard deviation	2797	2135
	Range	423–133,504	537–133,504

^a^Applied Mann-Whitney test. Difference between groups statistically significant (*p* < 0.001)

Baseline model (model 1) results presented in Table 4 show that the presence of diabetes increases the chance of multiple admissions for any ACSC by 1.49 times, adjusting for age and sex. The chance of multiple admissions increases by 1.13 times for males and rises with age.

**Table 4:** Analysis of the association between diabetes and multiple admissions for ACSC over age (*n* = 301,334)

	(Model 1)	(Model 2)
Gender		
Female	0.885***	0.889***
	(0.0111)	(0.0111)
Age Group		
40–64	1.661***	2.083***
	(0.0404)	(0.0493)
65–80	2.387***	3.269***
	(0.0388)	(0.0471)
80+	2.742***	3.785***
	(0.0382)	(0.0462)
Interaction between age group and diabetes		
Diabetes diagnosis (=1)	1.492***	4.084***
	(0.0110)	(0.0817)
40–64 # diabetes diagnosis (=1)		0.439***
		(0.0867)
65–80 # diabetes diagnosis (=1)		0.352***
		(0.0837)
80+ # diabetes diagnosis (=1)		0.336***
		(0.0831)
Constant	0.359***	0.266***
	(0.0378)	(0.0458)

Robust standard errors in parentheses

*p < 0.05, **p < 0.01, ***p < 0.001

Assuming that diabetes effect varies over age, we have found that the presence of diabetes was associated with an increased risk of multiple use in younger ages relatively to older ages as shown in model 2 presented in Table 4. Patients aged 18–39 years old with diabetes are 4.08 times more likely to become multiple users than patients with the same age but without diabetes. Additionally, the increased chance of multiple admissions among diabetic patients was 1.79 times higher for patients aged 40–64 years old, 1.44 times for patients aged 65–80 years old and 1.37 times for patients aged 80 or older.

These results confirm the advanced hypothesis that the association between diabetes and the chance of multiple admissions by any ACSC is not constant across the age groups, once the interaction coefficients are significative.

The sensitive analysis, adjusted for gender and age group, allowed us to verify that, although the risk of multiple avoidable admission for diabetes slightly reduces when only three of the most prevalent avoidable conditions (bacterial pneumonia, heart failure and UTI) are analysed, the trend of increased risk of multiple avoidable admission by the presence of diabetes remains (Odds Ratio = 1.40). The table with the analysis can be found on Additional file 2.

## Discussion

In this study, we analysed the association between diabetes and multiple admissions for ACSC in Portugal. From 2013 to 2015, ACSC were responsible for 15.3% of all admissions to all public Portuguese NHS hospitals inpatient services. Approximately 41% of multiple ACSC admissions had a diabetes diagnosis, corresponding to 59,436 admissions, associated to 23,692 patients. On average, the length of stay was longer on admissions with diabetes (11 ± 12 days), when comparing with non-diabetes (10 ± 9 days), and medical expenditures were, on average, higher on admissions with diabetes (2543 ± 2797€), compared with non-diabetes (2261 ± 2135€). Causes of multiple admissions differ by age categories and by the presence of diabetes. Patients with diabetes were 1.49 times (*p* < 0,001) more likely to be admitted multiple times for any ACSC than non-diabetic patients. The presence of diabetes was associated with an increased risk of multiple use in younger ages relatively to older ages.

Admissions with diabetes diagnosis had a higher length of stay and higher costs per admission. This finding is in agreement with the literature confirming that diabetes contributes to a longer hospital stay [28], which consequently increases the average cost per admission.

In our study, the most prevalent ACSC was bacterial pneumonia, heart failure and UTI even on admissions with a diabetes diagnosis, which is consistent with results found by Kim et al. [29]. Our analysis showed that the cause of admission varies across age groups and with the presence or absence of diabetes. At older ages, the most prevalent causes of admission, by diabetic patients, were no longer those that directly related to diabetes but other conditions such as bacterial pneumonia and heart failure. We hypothesize that this variation is due to a higher burden of multimorbidity at older age groups [30]. A more in-depth study of the interaction between diabetes and other specific ACSC is needed to better understand their relationship with the likelihood of potentially avoidable admissions across the various age groups.

Younger diabetic patients had a relative higher risk of being multiple users of ACSC than those at older age groups. An explanation for this finding might be related to a worse glycemic control in younger patients and a possibly higher proportion of type 1 diabetes in this group. Berkowitz, Meigs and Wexler found that, even though people younger than 65 years have fewer comorbid conditions, they also have inferior glycemic control [31]. This may be explained by the social characteristics of this age group. Younger adults may be more focused on developmental tasks, such as employment, than on the management of their disease [32]. Poor health habits, such as malnutrition (high intake of trans fats), decreasing physical activity, alcohol and smoking consumption and high levels of stress may be some of the factors that difficult the management of the disease [32]. Another possible explanation for this finding may be related to differences in the pathophysiology of diabetes between older and younger adults [33].

Our results may also be interpreted by a healthcare system organization point of view. The occurrence of potentially avoidable admissions, which had the potential to be prevented through outpatient care, warns for some system inefficiency, but the recurrence of these admissions possibly points out systemic problems in responding to the needs of individuals. Therefore our results may reinforce the potential for improvement in the management of chronic diseases, such as diabetes, through quality outpatient care. Health care management strategies, particularly in a context of increasing complexity, are needed due to the increased prevalence of individuals with multiple chronic diseases and comorbidities, but also due to the need to contain costs in the area of health care.

There are limitations to our study. First, admissions for ACSC identified in the present study are only suggestive of potentially avoidable admissions since some of the admissions were necessary and could not have been avoided even with optimal outpatient care, once the natural worsening of the health status of individuals with chronic diseases can motivate and justify admission [34], so we reckon the possibility of overestimating the number of potentially avoidable admissions. Additionally, we used only administrative data which provide a set of relevant representative and appropriate information of the study of this phenomenon but has a set of well-known limitations related to the quality of the information, in particular regarding the codification of diagnosis [35]. Although we considered a set of control variables related to health status and demographic aspects, namely sex, age group and CCI, we acknowledge that it could be interesting to analyse socioeconomic and provision of health services variables since some studies concluded that those variables have an association with ACSC admissions.

## Conclusions

In conclusion, our results demonstrate a relevant association between diabetes, a chronic and complex disease, and the occurrence of multiple admissions for ACSC.

The findings highlight the need to define and prioritize a set of strategies, structured, both locally and organizationally, specially oriented to younger adults such as health promotion and health education, to improve the management of chronic diseases and reduce its consequences. Notwithstanding the contributions of the present study, we emphasize that future research is important, not only to better understand this phenomenon and its relation with other diseases, but also to define and implement effective strategies in order to reduce potentially avoidable admissions and their multiplicity.

## Supplementary information

**Additional file 1.** Composite Prevention Quality Indicators (PQIs) – PQI 90 Overall composite.

**Additional file 2: Table S4.** Logistic regression sensitivity analysis of the 3 most prevalent and non-related diabetes avoidable conditions, adjusted odds ratios (AOR) by sex and age group.

## Data Availability

The data that support the findings of this study are available from Administração Central do Sistema de Saúde (ACSS), I.P., but restrictions apply to the availability of these data, which were used under license for the current study, and so are not publicly available. Data are however available from the authors upon reasonable request and with permission of Administração Central do Sistema de Saúde (ACSS), I.P.

## Abbreviations

ACSC: Ambulatory care sensitive conditions
AHRQ: Agency for Healthcare Research and Quality
CCI: Charlson Comorbidity Index
COPD: Chronic Obstructive Pulmonary Disease
DRG: Diagnosis Related Groups
ICD-9-CM: International Classification of Diseases, Version 9 – Clinical Modifications
NHS: National Health Service
PQIs: Prevention Quality Indicators
UTI: Urinary Tract Infections
