# Supplementary material for: The impact of diabetes on multiple avoidable admissions: a cross-sectional study
Source: BMC Health Serv Res. 2019 Dec 27;19:1002. doi: 10.1186/s12913-019-4840-4 (PMC6935195; doi:10.1186/s12913-019-4840-4)
Supplement: Supplementary file 1 — Additional file 1. Composite Prevention Quality Indicators (PQIs) – PQI 90 Overall composite. [file 12913_2019_4840_MOESM1_ESM.docx]

**Supplementary Data I**

*Composite Prevention Quality Indicators (PQIs) – PQI 90 Overall composite*

| PQI 01 – Diabetes short-term complications admission rate;  PQI 03 – Diabetes long-term complications admission rate; |
| --- |
| PQI 05 – Chronic Obstructive Pulmonary Disease or Asthma in Older Adults Admission Rate; |
| PQI 07 – Hypertension Admission Rate; |
| PQI 08 – Heart Failure Admission Rate; |
| PQI 10 – Dehydration Admission Rate; |
| PQI 11 – Bacterial Pneumonia Admission Rate; |
| PQI 12 – Urinary Tract Infection Admission Rate; |
| PQI14 – Uncontrolled Diabetes Admission Rate; |
| PQI 15 – Asthma in Younger Adults Admission Rate; |
| PQI 16 – Lower-Extremity Amputation among Patients with Diabetes Rate |

**Source:** (AHRQ 2016)
