# Supplementary material for: The impact of diabetes on multiple avoidable admissions: a cross-sectional study
Source: BMC Health Serv Res. 2019 Dec 27;19:1002. doi: 10.1186/s12913-019-4840-4 (PMC6935195; doi:10.1186/s12913-019-4840-4)
Supplement: Supplementary file 2 — Additional file 2: Table S4. Logistic regression sensitivity analysis of the 3 most prevalent and non-related diabetes avoidable conditions, adjusted odds ratios (AOR) by sex and age group. [file 12913_2019_4840_MOESM2_ESM.docx]

**Supplementary Data II**

***Table S4.*** *Logistic regression sensitivity analysis of the 3 most prevalent and non-related diabetes avoidable conditions, adjusted odds ratios (AOR) by sex and age group.*

|  | **Multiple ACSC admissions for bacterial pneumonia, heart failure and UTI** |
| --- | --- |
|  | **AOR [IC 95%]** |
| Diabetes* | **1.401** (1.376 – 1.427) |
| **Male sex** | **1.069** (1.051 - 1.087) |
| **Age Group**  18 – 39 (reference)  40 – 64  65 – 79  ≥80 | **1.000**  **1.695** (1.587 – 1.811)  **2.830** (2.658 – 3.014)  **3.416** (3.211 – 3.634) |

*Statistically significant (p<0.01)
